# Barriers and Opportunities in Cancer Pain Management: A Qualitative Study on Pharmacists’ Role

**DOI:** 10.3390/pharmacy13060173

**Published:** 2025-12-01

**Authors:** Evangelos Aliferis, George Koulierakis, Christina Dalla, Tina Garani-Papadatos

**Affiliations:** 1 Department of Public Health Policy, School of Public Health, University of West Attica, 11521 Athens, Greecesgarani@uniwa.gr (T.G.-P.); 2 2nd Department of Obstetrics-Gynecology, Aretaieio Hospital, Medical School, National and Kapodistrian University of Athens, 11521 Athens, Greece

**Keywords:** cancer pain, pharmacists, palliative care, interdisciplinary collaboration, right to pain-relief, supportive oncology

## Abstract

**Introduction:** Cancer pain remains a critical issue for patients’ quality of life, affecting their physiology, psychology, and social relationships. Despite the widely recognized role of pharmacists in pain management, their involvement in palliative care in Greece remains limited. This study focuses on exploring the perceptions and experiences of pharmacists regarding their role in cancer pain management, identifying barriers, required skills, and proposing strategies for their integration in the multidisciplinary team. **Μaterials and Μethods:** Qualitative research was conducted through semi-structured interviews with seven pharmacists in the Attica region. The interviews were recorded, transcribed, and thematically analyzed. **Results:** The analysis revealed four main themes: (1) limited access to medical records and challenges in pharmaceutical decision-making, (2) lack of institutional frameworks and a culture of collaboration, (3) need for specialized education and continuous training, and (4) understaffing and bureaucracy, faced by pharmacists. **Discussion:** This study highlights the underutilized role of pharmacists in cancer pain management in Greece. Barriers such as restricted access to patient records, weak interdisciplinary collaboration, insufficient training, and bureaucratic constraints limit their contribution. Structured frameworks and collaborative cultures can enhance pharmacists’ involvement, while education and continuous training are essential to strengthen their legitimacy within care teams. Digital tools can improve access to patient information and support evidence-based decisions. **Conclusions:** Pharmacists’ integration in the patient’s management team has significant benefits for the patient’s quality of life. Strengthening pharmacists’ involvement in cancer pain management requires the establishment of collaborations, continuous education, bureaucratic simplification, and the integration of digital tools. The development of practical resources, such as educational guides, can play a pivotal role in enhancing the quality of care provided.

## 1. Introduction

With rising cancer incidence rates and evolving treatment modalities, patients are increasingly living longer while continuing to experience cancer-related pain. This shift highlights pain as a persistent clinical feature that significantly disrupts the lives of both patients and their families [1]. Despite considerable advancements in science and technology, cancer remains one of the leading causes of death globally (World Health Organization, 2021) [2]. The high prevalence of cancer cases renders the issue of cancer pain management particularly critical for public and community health systems [3].

Whether originating from metastatic disease, surgical procedures, or therapeutic interventions such as radiotherapy [4], cancer pain has been characterized as total pain, as it permeates all dimensions of human existence [5], affecting not only the patient’s physiology but also their dignity, psychological and spiritual well-being, as well as social life [6,7]. Effective pain management is essential. It encompasses self-management when clinically appropriate, support from family and caregivers, and, most importantly, care delivered by qualified healthcare professionals [8]. The absence of relief and the persistent experience of suffering act as major obstacles to the realization of the fundamental human right to be free from pain, particularly in terminal stages [9].

Within this context, the present research is grounded in the observation that, although the pharmacist’s role in pain relief has been internationally recognized [10], pharmacists in Greece are either not involved or only minimally engaged in the pharmaceutical management of cancer pain and the provision of palliative care. Although pharmacists’ contribution to cancer pain management has been increasingly recognized in several international contexts, such as the United Kingdom and Japan, where structured frameworks and interdisciplinary collaboration are widely established, the Greek setting remains largely unexplored. To date, no qualitative study has systematically examined pharmacists’ perceptions and experiences in palliative care within Greece. This absence represents a significant gap in the literature, particularly given the distinctive institutional and cultural features of the Greek healthcare system, including limited access to medical records, hierarchical decision-making, and regulatory constraints. Addressing this gap is critical, as regional originality not only strengthens the evidence base but also provides insights that may inform national policy and contribute to the broader international discourse on pharmacist integration in cancer pain management.

The aim of the present study is to explore the perceptions, practices, and experiences of Greek pharmacists concerning the management of cancer-related pain and the delivery of palliative care. Study objectives include identifying primary barriers to pharmacists’ engagement in palliative care, documenting essential skills and knowledge required to enhance their role, and formulating recommendations to improve existing practices and overall integration of pharmacists into cancer pain management and palliative care.

## 2. Materials and Methods

### 2.1. Research Approach

This study adopts a qualitative research methodology including semi-structured face-to-face interviews to describe, interpret, decode, and derive meaning from a given phenomenon [11]. Τhe study incorporates elements of phenomenology, as it investigates the participants’ personal and social experiences [12,13]. This approach is deemed as the most appropriate for exploring complex social phenomena, such as pharmacists’ attitudes and involvement in cancer pain management.

### 2.2. Participants

To be eligible for inclusion, pharmacists were required to have a minimum of five years of professional experience. No additional exclusion criteria were applied. While participants were not required to work exclusively in cancer care, their professional background ensured relevant exposure to pharmaceutical practice. Recruitment continued until thematic saturation was reached.

A purposive sampling strategy was utilized for participant recruitment, specifically employing the snowball sampling technique. Initially, participants were contacted by e-mail through the first author’s professional network. After agreeing to participate, they referred to additional individuals who matched the desired selection criteria. Those who agreed to participate met with the interviewer who provided detailed information about the study and obtained written informed consent.

The study was conducted in community pharmacy settings within the capital region, rather than a single institution.

Seven pharmacists participated in the study. Their ages ranged from 34 to 53 years, and their professional experience spanned from 10 to 25 years. The sample included three women and four men. Participant characteristics are summarized in Table 1. Sample adequacy was determined according to the principle of thematic saturation. Saturation was reached when successive interviews no longer yielded new codes or themes relevant to the research questions. Specifically, after the sixth interview, no additional substantive codes emerged, and the seventh interview confirmed the stability of the thematic categories.

### 2.3. Interview Guide

Data was collected through semi-structured, face-to-face interviews, which were guided by an interview protocol developed after a review of the relevant literature [4]. which was conducted using systematic searches of electronic databases (e.g., PubMed, Scopus, Web of Science) to identify both existing and current studies relevant to pharmacists’ roles in cancer pain management and palliative care. Keywords included “pharmacist,” “cancer pain,” “palliative care,”. The interviews were conducted by the first author (EA, Pharmacist) between July and December 2024, at a time and location chosen by each participant. The average duration of the interviews was 20 min. All interviews were audio-recorded, anonymized, and subsequently transcribed verbatim by the interviewer. The transcriptions were reviewed by the interviewer and the last author against the audio recordings to ensure accuracy. No follow-up interviews were conducted.

### 2.4. Procedure

Prior to the interview, an informal conversation was held with each participant to establish rapport and build a sense of trust. The interviewer explained the study’s objectives and provided detailed information regarding the interview content, with particular emphasis on data protection, the right of participants to withdraw from the study at any time, and the possibility to ask questions. Participants were invited to describe their experiences in their professional setting, as well as the state of pharmaceutical practice, interdisciplinary collaboration, and education in relation to cancer pain management and palliative care provision.

### 2.5. Data Analysis

The analysis was based on an inductive approach and employed thematic analysis as the primary method [13,14]. All recorded interviews were first transcribed into written form, preserving critical details and non-verbal cues, such as pauses and tone of voice. The transcripts were repeatedly reviewed to ensure data familiarization, and notes were made regarding points of notable significance. Subsequently, coding of the data was carried out, organizing content into smaller units and categories that reflected key meanings and concepts. Each paragraph was examined in detail to identify words or phrases relevant to the research focus, generating codes such as “perception of the pharmacist’s role” and “barriers to participation.” Related codes were grouped and organized into thematic clusters. This process led to the identification of key themes and subthemes aligned with the research questions. Themes were further reviewed to confirm their alignment with the data, and modifications or consolidations were made to ensure coherence and relevance. The findings are presented as themes and subthemes, supported by examples and representative excerpts from the interviews to substantiate the conclusions. The methodological reporting and presentation of the results conform to the Consolidated Criteria for Reporting Qualitative Research (COREQ) [15].

### 2.6. Ethical Considerations

The study received approval from the Research Ethics and Deontology Committee of the University of West Attica (UniWA—Protocol No.: 50921—27 June 2024).

## 3. Results

The analysis of the interview data yielded four overarching themes, each comprising specific subthemes: (1) Pharmaceutical Approach, (2) Interdisciplinary Collaboration, (3) Education, and (4) Limiting Factors (Table 2).

### 3.1. Pharmaceutical Approach

This theme pertains to the participants’ views regarding pharmacists’ access to information related to the patient’s complete pharmacological profile. Participants emphasized that limited access to full medical records represents a key barrier to strengthening their role.

*“…No, we don’t have access to details or the patient’s medical history. Only the attending physician has such access…”* (Peter—pseudonym)

They highlighted the pharmacists’ inability to provide optimal pharmaceutical services without comprehensive information. The need to improve access to medical data was a recurrent theme and was directly associated with treatment safety and effectiveness, as pharmacists’ capacity to make informed, autonomous decisions is hindered by the absence of access to the patient’s medical file.

*“…The lack of access to full medical records makes it difficult to check for interactions. We often rely on incomplete data…”* (Helen—pseudonym)

*“…We don’t have the necessary information to intervene effectively in pharmacotherapy. Without a complete picture of the patient’s history, our capacity to contribute is limited…”* (Antony—pseudonym)

Enhancing digitalization and interconnectivity of healthcare information systems were identified as factors which could substantially improve pharmacists’ access to comprehensive pharmacological histories.

*“…Utilizing technology would certainly be a positive development. The digitization of medical files and information-sharing among healthcare professionals—although it seems difficult to implement under current conditions—would facilitate many aspects of care and potentially resolve some persistent issues…”* (Nick—pseudonym)

### 3.2. Interdisciplinary Collaboration

Participants underscored the importance of interdisciplinary collaboration for improving cancer pain management and pointed out the absence of an institutional multidisciplinary framework, clarifying that team structures should expand to include pharmacy representation. Although they pointed to difficulties in establishing cooperation with physicians, they did not mention problems in collaboration with nurses or other specialties involved. The focus on collaboration with physicians and the lack of mention to other specialties by the interviewees can be considered as a finding of this study, in line with the primary position of the attending physician in the care of the patients.

*“…There is no formal framework… Ideally, collaboration with the attending physician and close coordination with the pharmacist should exist. But it doesn’t… I’ve encountered physicians who are cooperative, and others who say ‘I can manage my patient on my own’… Without formalized structures, pharmacists are left without the support they need…”* (Maria—pseudonym)

They also noted significant potential for improvement in interdisciplinary collaboration, which could foster better communication among healthcare professionals and ultimately enhance patient quality of life.

*“…Collaboration among various disciplines is essential for holistic patient care. If there were a structured framework to promote this collaboration, patients would benefit from a more coordinated approach to pain management…”* (Anastasia—pseudonym)

Nonetheless, pharmacists reported very limited collaboration, particularly with attending physicians.

*“…We only contact the physician in cases of glaring mistakes—otherwise, they typically won’t accept our input…”* (Antony—pseudonym)

*“…That sort of collaboration doesn’t exist in our pharmacy or hospital—and I believe it’s generally absent across the Greek healthcare system. We can’t say there’s a culture of collaboration…”* (Anastasia—pseudonym)

*“…Effective cooperation isn’t only a matter of individual effort—it’s also a question of collective institutional culture within a healthcare setting…”* (Maria—pseudonym)

Therapeutic regimen management emerged as a friction point in interdisciplinary collaboration, with pharmacists expressing a desire for greater participation in clinical decision-making. These testimonies suggest that the leading role of physicians within healthcare settings may limit pharmacists’ involvement, with physicians often making independent decisions regarding dosing and therapeutic approaches.

*“…It’s not easy to suggest changes to a treatment regimen already chosen by a physician…”* (Helen—pseudonym)

*“…Physicians decide on the dosage on their own; we’ve never been asked to contribute our opinion…”* (Peter—pseudonym)

*“Most of the time, the physician is considered the sole authority…”* (Anastasia—pseudonym).

The absence of well-defined frameworks for collaboration can limit the effectiveness of patient care. Pharmacists often experience a sense of working independently, while variations in physicians’ perspectives toward other specialties underscore the importance of fostering a collaborative culture. This may be supported through focused educational efforts and institutional improvements.

### 3.3. Education

Specialized education was recognized as a fundamental factor in strengthening the role of pharmacists within the multidisciplinary healthcare team. Participants emphasized the importance of ongoing training in pain management.

*“…From my experience, if you demonstrate scientifically sound knowledge with seriousness and supporting evidence, the physician will listen—and even if not the first time, they will the second. We can build this ourselves…”* (Anastasia—pseudonym)

*“…This could be achieved through an update of our knowledge. We could become more accepted and respected by other healthcare professionals and even by patients…”* (Anastasia—pseudonym)

Participants highlighted education as the foundation for meaningful clinical participation by emphasizing that specialized training in pain management with added emphasis in cancer pain, given its high prevalence, is essential for establishing pharmacists’ legitimacy within the clinical team.

*“…With proper training and the use of technology, the pharmacist could assume a meaningful role in pharmaceutical schemes and pain management…”* (Aris—pseudonym)

*“…To participate equally in a team, you must be able to substantiate your role. Education is the starting point for that process…”* (Maria—pseudonym)

*“…The clinical pharmacist needs to have subject-specific expertise. Within the hospital setting—will they go to the oncology department? Hematology? The ICU?…”* (Anastasia—pseudonym)

Pharmacists considered further education essential for addressing complex pain management cases. Their recognition of this need reflects a strong commitment to improving their competencies and the establishment of targeted educational programs was viewed as catalytic for enabling their equal participation in patient care.

*“…Pain management is complex, and as pharmacists, we need more specialized training. Without continuous professional development, our contribution remains limited…”* (George—pseudonym)

Participants supported the development of a comprehensive reference guide that would offer practical and educational support on general topics related to palliative care, even from undergraduate education onward. The proposal to create such guides and educational materials highlights the need for practical support and the strengthening of pharmacists’ confidence and capacity

*“…Such a guide would be extremely useful and would empower pharmacists even without prior specialized training in the field…”* (Nick—pseudonym)

Participants also underscored the role of new technologies, whose relevance emerged clearly during the interviews.

*“…At first, new technologies might present difficulties—but ultimately, they’re always helpful and elevate the services we can provide…”* (Aris—pseudonym)

*“…I believe new technologies would greatly assist both the pharmacist and the patient. Digitization of information would significantly facilitate our work…”* (Helen—pseudonym)

### 3.4. Limiting Factors

Participants identified various constraints—including restrictive legislation, bureaucratic processes, understaffing, and high workload—that hinder their effectiveness in pain management.

*“…There are far too few hospital pharmacists, all carrying overwhelming workloads. That is a major obstacle…”* (Nick—pseudonym)

*“…Nationwide, in practically every hospital pharmacy, the number one issue is understaffing. This results in multiple responsibilities per person and sometimes even becomes a pretext for avoiding engagement in additional tasks…”* (Maria—pseudonym)

Strict regulations governing the prescribing and dispensing of pain-related medications—combined with bureaucratic hurdles such as excessive paperwork, multi-step approval processes for opioids, and fragmented communication channels—significantly constrain pharmacists’ ability to manage cancer pain effectively.

*“…As pharmacists we have so many responsibilities in our daily workflow that our ability to dedicate time to individualized pain management is restricted…”* (Maria—pseudonym).

To further illustrate the barriers identified through the interviews, Table 3 summarizes pharmacists’ perceived obstacles to collaborative cancer pain management and highlights their direct consequences for clinical practice and patient care.

## 4. Discussion

This qualitative study is the first in Greece aiming to explore pharmacists’ perspectives on their role in addressing cancer-related pain. Participants articulated both the underutilization of their professional expertise and the challenges hindering their active involvement in cancer pain management and the provision of palliative care. A primary concern raised in the interviews was the limited access to comprehensive patient pharmacological information. Although pharmacists require full and updated medical histories and treatment plans to make evidence-informed decisions that benefit patients [16], their access to such data remains highly restricted.

Also noteworthy was the participants’ positive attitude toward the integration of new technologies, which are viewed as essential tools for enhancing efficiency and quality of care. While initial implementation may be demanding, the digitization of information and deployment of innovative technologies offer considerable advantages, including improved data organization, faster communication, and greater accuracy in pharmacological interventions, thus minimizing errors. Moreover, the use of advanced software systems enabling access to clinical data could significantly optimize pharmaceutical care delivery [17]. While participants expressed strong support for digital health solutions, it is important to acknowledge potential barriers to their implementation within the Greek healthcare system. Data privacy concerns remain a critical issue, particularly given the sensitive nature of cancer patients’ medical records and the need for compliance with European and national regulations such as the General Data Protection Regulation (GDPR) and the corresponding national legislation. In addition, interoperability challenges between hospital information systems and community pharmacies may hinder seamless data exchange, limiting the effectiveness of digital integration. Legislative constraints, including restrictive prescribing and documentation requirements, further complicate the adoption of digital platforms. Recognizing these realities underscores our findings that adds depth and practical realism to our findings, underscoring that while digital tools hold significant promise, their successful integration requires parallel policy reforms, infrastructure investment, and robust safeguards to ensure patient trust and system efficiency. Therefore, the introduction of digital tools and data management platforms could empower pharmacists with critical information, enhancing both the accuracy and timeliness of their decisions and contributing to greater safety and quality of care.

Participants also emphasized the need for improved interdisciplinary collaboration. Pharmacists face significant barriers when attempting to integrate into oncological care teams, resulting in limited communication and cooperation with other healthcare professionals [18]. This fragmentation undermines the full utilization of pharmacists’ pharmacotherapeutic expertise, particularly as far as the management of analgesics, opioids, and anticancer medications are concerned. The same applies to addressing specific patient needs such as difficulty of a patient in swallowing oral formulations, adverse effects or drug interactions. and through patient and caregiver education, provided by the pharmacists, can lead to meaningful benefits for patients, families, teams, and institutions [19,20].

One of the most prominent issues raised by the participants was the lack of specialized training among pharmacists in oncology and pain management. Palliative care requires in-depth knowledge and a nuanced understanding of the needs of patients with cancer, and insufficient training can lead to suboptimal care outcomes [21]. The development of continuing education programs and targeted seminars could equip pharmacists with the specialized skills and knowledge necessary for effective contribution [22]. Simultaneously, the integration of relevant content into undergraduate pharmacy curricula would lay the foundation for a more comprehensive approach to pain management.

Restrictive regulations and bureaucratic procedures were also identified as key obstacles that have been documented internationally [23]. Regulatory reforms related to the prescribing and dispensing of medications—particularly controlled analgesics—as well as streamlining administrative processes, could facilitate pharmacists’ engagement in cancer pain management and enhance their integration into palliative care services. This, in turn, would enable a stronger focus on patient-centered needs [24].

Participants further reported high workloads and time constraints frequently hinder their active participation in interdisciplinary care teams. Recruiting additional staff, especially in hospital pharmacies, along with more efficient task distribution, could alleviate these pressures [22]. In a related study conducted in Japan, 70% of pharmacists reported some involvement in palliative care teams, while 16% indicated no involvement, citing lack of time (90%) and understaffing (68%) as the main reasons [25]. Understaffing and work overload appear to be critical barriers to care delivery, whereas strengthening human resources could substantially elevate the professional contribution of pharmacists.

Our findings resonate with international experiences but also highlight context-specific barriers in Greece. For example, in the United Kingdom, pharmacists are increasingly integrated into palliative care teams, supported by national frameworks that encourage interdisciplinary collaboration and shared access to patient records. Similarly, in Japan, surveys have shown that a majority of hospital pharmacists participate in palliative care, though challenges such as understaffing and time constraints remain. By contrast, the Greek context is characterized by limited institutional structures and hierarchical decision-making, which restrict pharmacists’ involvement despite their willingness and expertise. Conceptually, these barriers can be understood as the product of systemic factors—such as regulatory rigidity, insufficient workforce resources, and a medically oriented decision-making system—that hinder the translation of pharmacists’ real potential into practice. Situating our results within these broader frameworks underscores both the horizontal character universality of certain challenges and the unique institutional dynamics that explain their persistence in Greece.

When asked about the potential positive effect of a structured guidance framework, including basic scientific and ethical principles regarding pain management for cancer patients, all participants had a positive reaction, stating that such an educational and guiding tool would indeed contribute to the enhancement of the pharmacists’ role, providing a solid knowledge base and evolving dynamically in response to emerging clinical needs and scientific evidence. Developing a similar instrument would be in line with international initiatives which currently aim to improve cancer pain management while promoting the integration of pharmacists in this context. The World Health Organization (WHO), for instance, has published pharmacologic and radiotherapeutic guidelines for cancer pain, offering practical recommendations for healthcare providers, including pharmacists, to ensure effective pain relief. Likewise, the National Comprehensive Cancer Network (NCCN) has developed clinical guidelines that, in addition to personalized recommendations for the use of analgesics and opioids, strongly advocate for interdisciplinary collaboration.

These initiatives suggest that the development of guiding and educational material for pharmacists involved with cancer patients could draw upon the best international practices and be tailored to local healthcare needs, thereby reinforcing the pharmacist’s role in supporting patients experiencing cancer-related pain. Participants underscored the necessity of establishing a structured, team-based framework for cancer pain management and palliative therapy, recognizing it as a strategic priority to reinforce and expand the pharmacist’s role. International experience likewise highlights the importance of collaborative care models, where pharmacists are fully integrated into multidisciplinary teams, ensuring that their expertise contributes to optimized medication management, patient support, and the overall quality of palliative care. Its creation would represent a strategic step toward enhancing the presence of pharmacists in palliative care, and its adaptability would serve as a cornerstone of its long-term effectiveness. Moreover, through coordinated efforts involving professional and institutional bodies, this roadmap could yield a substantial improvement in the quality of care provided to patients living with cancer pain.

## 5. Conclusions

Pharmacists’ involvement in palliative care remains both limited and underexplored, a fact which stands in contrast to emerging international practices. Nonetheless, improvement in their underutilization may be achievable through targeted interventions. Continuing education, promotion of collaborative practice, and access to complete patient medical records are essential for advancing this objective. Pharmacists are well-positioned to play a vital role in enhancing the quality of life of cancer patients through more effective pain management. Practical tools—such as the development of a dedicated clinical guide (defined as a structured, evidence-based manual offering standardized protocols and practical decision-making support for pharmacists in palliative care)—could support this goal.

### Strengths and Limitations

A key strength of this study is for the first time in Greece it investigates a sensitive issue of utilization of a health profession’s potential for the benefit of the community, and offers a comprehensive depiction of the current state regarding the pharmacists’ participation and role in the relief of cancer-related pain in terminal-stage patients. As with all qualitative research, a primary limitation lies in the limited generalizability of the findings, due to the use of small, non-random samples that may not be representative of larger populations. Additionally, qualitative studies are inherently subject to interpretation bias, as the researcher’s perspective may influence the analysis and interpretation of the data.

## Figures and Tables

**Table 1:** Socio-demographic characteristics of the participants.

ID	Age	Gender	Professional Experience (Years)	Education Level	Professional Title
1	41	Male	15	Postgraduate	Community Pharmacist
2	47	Male	25	Postgraduate	Hospital Pharmacist (Director)
3	35	Male	10	Postgraduate	Hospital Pharmacist
4	38	Male	12	Postgraduate	Hospital Pharmacist
5	53	Female	25	Postgraduate	Hospital Pharmacist (Director)
6	48	Female	25	Postgraduate	Hospital Pharmacist (Director)
7	35	Female	13	Postgraduate	Hospital Pharmacist

**Table 2:** Thematic Categories and Subtheme Analysis.

Pharmaceutical Approach	Interdisciplinary Collaboration	Education	Limiting Factors
Limited medical records access	Lack of formal frameworks	Need for specialized training	Understaffing, bureaucracy
Incomplete data	Weak Collaboration culture	Value of scientific evidence	Strict prescribing rules
Autonomy constraints	Difficulty changing protocols	Pain/oncology as training priorities	Time limits, multiple duties

**Table 3:** Pharmacists’ Perceived Barriers to collaborative cancer pain management.

Barrier	Consequence
Medical personnel	
Lack of collaboration culture	Pharmacists left working in isolation
Physician dominance in decisions	Emotional and professional discouragement
Limited multidisciplinary participation	Reduced patient benefit
Health system	
Heavy workload	Multiple duties hinder team integration
No access to patient records	Inability to provide optimal pharmaceutical care
Bureaucracy	Very limited time for collaborative practices
Knowledge	
Limited digital skills	Lower efficiency in care delivery
Lack of subject-specific expertise	Reduced quality of care

## Data Availability

The data presented in this study are available on request from the corresponding author due to privacy and ethical restrictions.
